# Addictive disorders, psychiatric symptoms, and potentially traumatic events in individuals with disabilities

**DOI:** 10.3389/fpsyg.2022.936184

**Published:** 2022-10-13

**Authors:** Rodrigo Marín-Navarrete, Ricardo Sánchez-Domínguez, Alejandro Pérez-López, Ricardo Saracco-Alvarez

**Affiliations:** ^1^Division of Research and Translational Education, Centros de Integración Juvenil, Mexico City, Mexico; ^2^Division of Clinical Research, Instituto Nacional de Psiquiatría Ramón de la Fuente Muñiz, Mexico City, Mexico

**Keywords:** individuals with disability, addictive disorders, psychiatric disorder, potentially traumatic events, unmet needs

## Abstract

**Background/Objectives:**

Individuals with disabilities (IWD) have a higher risk of potentially traumatic events (PTEs) either in childhood or adulthood, increasing the risk of suicide attempts, mental disorders, and substance use disorder. The aim of this study was to explore the association between substance use, psychiatric symptoms and suicidal behavior with PTEs. A Multisite cross-sectional study was conducted.

**Materials and methods:**

The sample includes 1,098 participants with any type of disability (motor, intellectual, visual, and mixed) located in Mexico City. Traumatic events, violence, discrimination, addictive disorders, and psychiatric disorders were examined. Multivariate logistic regression models were conducted. Data was collected between September–October 2014.

**Results:**

People with motor or visual disability have a higher prevalence in nicotine use disorder (NUD), generalize anxiety disorder (GAD), mayor depression disorder (MDD), want to be dead, and lifetime suicide attempts. Intellectual disability group only presents GAD and MDD. All disability groups have a high prevalence of PTEs. Verbal violence in childhood, sexual abuse, discrimination and serious accidents had a strong impact in the development of NUD, psychiatric symptoms and suicidal behavior.

**Conclusion:**

These findings show the relevance of develop specific tools for detection, referral and treatment, in order to improve the mental health of people with disabilities.

## Introduction

Experiences throughout life can impact the mental health of people, previous evidence indicates that lifetime exposure to potentially traumatic events (PTEs) such as being involved in serious accidents, natural disasters, life-threatening illness, be witness of death or murders; physical or sexual abuse can cause a severe stress response, which could modify the perception of self-threat, and other factors such as resilience, vulnerability, negative emotions, and emotional dysregulation (Overstreet et al., 2017; Van der Velden et al., 2017). Several repots estimated that approximately the 80% of individuals in clinic samples and general population have been exposed to at least one lifetime PTEs (Norris et al., 2003; Dragan and Lis-Turlejska, 2007; Overstreet et al., 2017).

Diverse studies have associated the exposure to PTEs with substance use (Kilpatrick et al., 2003), psychiatric symptoms (Bandelow et al., 2005; Amstadter et al., 2013), and suicidal behavior (Bandelow et al., 2005). However, there are limited information about the exposure to PTEs in at-risk groups as individuals with disability (IWD) and its outcomes in mental health, showing the need for detection of possible cases, referral and treatment for professional management to specialized services (World Health Organization [WHO], 2011).

Studies show that IWD have greater exposure to PTEs, particularly physical and sexual abuse (Focht-New et al., 2008). Recent research has reported that mental health problems may be associated with verbal violence and discrimination due to the type of disability, causing interpersonal problems, academic, health problems, and unemployment (Li and Moore, 2001; Iezzoni, 2011).

Exposure to PTE in IWD can lead to the risk of developing substance use or substance use disorder (SUD; Didden et al., 2009), alcohol use disorder (AUD; Li and Moore, 2001), psychiatric symptoms (Sequeira and Hollins, 2003), and suicidal behavior (Lund et al., 2016; Lutz and Fiske, 2018). Previous evidence indicates that smoking prevalence in past month among IWD, oscillates between 7 and 20.5% (Steinberg et al., 2009), alcohol use ranges between 35 and 39% (Gress and Boss, 1996; Pack et al., 1997) and any substance use is reported in about 18% (McGillicuddy and Blane, 1999), although other studies suggest that any substance use could reach up to 50% (Smedema and Ebener, 2010). The most used substance among IWD is tobacco (Soule et al., 2015) followed by alcohol, cannabis, and cocaine (Chaplin et al., 2011; Glazier and Kling, 2013).

To our knowledge, this is first study carried out in Mexico focused in measure multiples variables associated to the mental health in IWD. Given this context, the aim of this study was to explore the association between PTEs with substance use, psychiatric symptoms and suicidal behavior among IWD.

## Materials and methods

### Study design

Multisite cross-sectional study.

### Participants

The sample was recruited through a non-probabilistic sampling. Participants were recruited from seven Community Development Centers (CDCs) for IWD located in Mexico City. During data collection, participants were receiving financial support from a government program for IWD. The inclusion criteria were: (a) people with motor, visual, intellectual, or mixed disability; (b) aged between 18 and 60 years old; (c) understand and sign an informed consent; and (d) to be beneficiaries of the governmental program for IWD. Exclusion criteria were (a) presenting a communication disability (difficulties to listen or speaking) and (b) not understanding informed consent.

**FIGURE 1:**
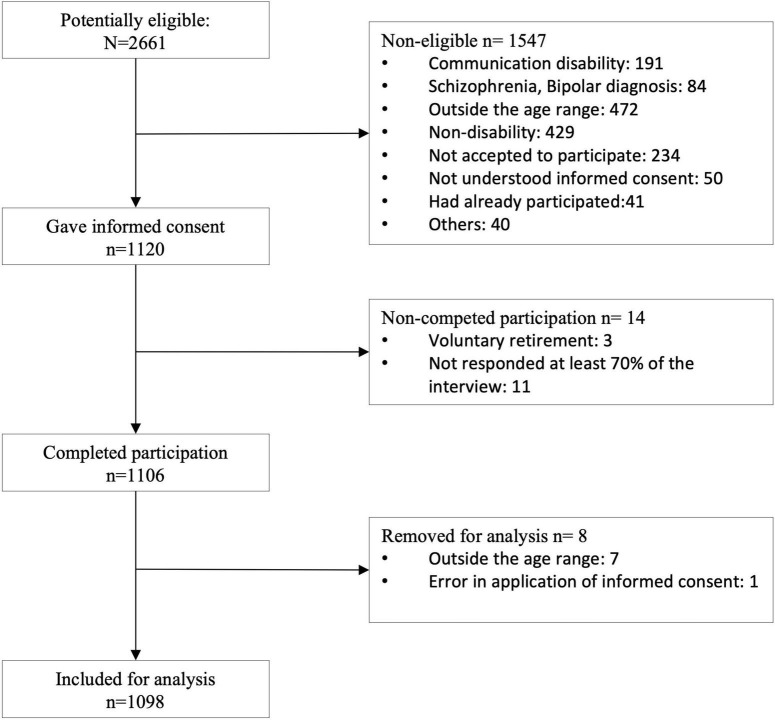
Participants flow chart.

Two thousand six hundred and sixty-one participants were recruited for this study but only one thousand and ninety-eight participants were included for analysis after eligibility, informed consent process and successfully completed participation (see Figure 1).

### Sites

Participant sites (called CDCs) aim to promote community development though free-time activities as sports, cultural activities, job training, and education. In addition, CDCs offers mental health and other health services to different population (Secretaria de desarrollo social [SEDESOL], 2005). CDCs are administered by System for Family Integral Development in Mexico City (DIF-CDMX).

### Measures

#### Disability

It was considered the disability classification used by DIF-CDMX. It includes motor, visual, intellectual, and mixed disabilities. The first refers to difficulties for walking, manipulating objects or moving. The second one refers to vision impairment (totally or partially). The third includes Down syndrome, dementia, and moderate/severe developmental delay. The latter combines at least two of the above categories.

#### Sociodemographic variables

Information was collected through a questionnaire, including demographic details, such as age, sex, years of education, current employment, and marital status.

#### Exposure to potentially traumatic events

In order to measure exposure to potentially traumatic events, the Brief Trauma Questionnaire (BTQ) was used. The BTQ is a 10-item instrument that measure lifetime exposure to 10 traumatic events according to the DSM-IV PTSD diagnosis (Schnurr et al., 2005). Responses are given as 0 (no) or 1 (yes) for each item. A score of 4 or greater on this scale indicate positive cases of PTSD with a sensitivity of 80%, specificity of 97%, positive predictive value of 71%, and negative predictive value of 98% (Breslau et al., 1999).

#### Interpersonal violence exposure

Violence was assessed through questions that covered the last 12 months (“¿Have you been insulted and made feel bad through shouting or offending?”, “¿Have you been threatened with being hit or hurt very hard?”). During childhood/adolescence (“¿Have you been forced to have sex or have sexual contact without your consent?”).

#### Perceived discrimination

Was measured with the question: “¿Have you ever felt discriminated due to your disability?” Response options were “yes, all the time,” “yes, quite often,” “yes, occasionally,” or “not.” Any affirmative answer was considered as perceived discrimination.

### Addictive disorders

#### Nicotine

Was measured with one question: “¿How many days did you smoke during the last 30 days?”, those who reported smoking 30 days were assumed to have a nicotine use disorder (NUD; Dierker et al., 2007).

#### Alcohol

Measure with the CAGE questionnaire. CAGE is composed by four items with dichotomy responses options (No = “0,” Yes = “1”; Ewing, 1984). CAGE has a high sensitivity and specificity for screening alcohol use disorder (AUD; Geneste et al., 2012). A cut-off score of 3 was considered AUD (Geneste et al., 2012).

#### Marijuana and cocaine

The Drug Abuse Screening Test (DAST) is a 20-item questionnaire used to assess substance use. Dichotomous responses are given as 0 (no) or 1 (yes) for each item (Gavin et al., 1989; Cocco and Carey, 1998). DAST has high sensitivity and specificity for screening drugs use disorder (Villalobos-Gallegos et al., 2015). For this study, a correlation item-total analysis was carried out. Items with a value higher than *r* = 0.70 were selected (items 8, 9, 11, and 12). Later, these items were tested with *ROC curves* identifying an area under the curve of 0.83. A cut-off score of 1 was utilized for screening substance use disorder (SUD).

#### Gambling

The Lie-bet Questionnaire (LBQ) is composed of two questions: “¿Have you ever had told important people to you how much you gambled?”, “¿Have you ever felt the need to bet more and more money?” LBQ has high sensitivity and specificity (over 80%) at a cut-off score of 1 for screening gambling problems (GD; Johnson et al., 1997).

### Psychiatric disorders

#### Generalized anxiety disorder

The questionnaire Generalized Anxiety Disorder (GAD-7) has seven items with response options scored from “0 = Not at all” to “3 = Nearly every day” (Kroenke et al., 2007). GAD-7 has a sensibility and specificity over 86.8% at a cut-off score of 10 for screening generalized anxiety disorder (GAD; García-Campayo et al., 2010).

#### Major depression disorder

The Patient Health Questionnaire (PHQ-9) has nine items whose responses options are scored from “0 = Not at all” to “3 = Nearly every day”. It has a sensibility and specificity over 89% (Diez-Quevedo et al., 2001; Baader et al., 2012). It consider a cut-off score of 10 for screening major depression (MDD; Kroenke et al., 2001).

#### Suicidal behavior

Was measured with the Mini-International Neuropsychiatric Interview (MINI v.5.0) suicide diagnosis. It has six items with dichotomy response options (No = 0, Yes = 1). This explore about suicidal ideation, plans and attempts during the last 30 days and lifetime (Lecrubier et al., 1997; Sheehan et al., 1998).

### Procedures

Participants were recruited in their respective centers (CDCs). Respondents who agreed to participate were evaluated to determine if they met the inclusion criteria, and subsequently signed an informed consent form. All participants included in this study gave written informed consent. In some cases, interviews were conducted in the presence of a significant other, but not facilitated by them. Interviews had an approximate duration of 40–60 minutes. Data collection took place between September–October 2014.

### Statistical analysis

Descriptive statistics were performed for demographic variables (age, sex, years of education, current employed). The One-way analysis of variance (ANOVA) was used to determine differences between disability groups (motor, visual, intellectual, and mixed) for numerical variables and *χ^2^* for categorical variables. To test the association between potentially traumatic events, independent multivariate logistic regression models were conducted. Model predictors were potentially traumatic events and the outcome of each criterion for nicotine use disorder, alcohol use disorder, gambling disorder, major depressive disorder, generalized anxiety disorder, past month death thoughts, suicide ideation, planning and attempt or lifetime suicide attempt. This procedure was carried out using *R* software v3.4.3, using the function *“glm”* from the package “*stats*.” A *p*-value < 0.05 was considered significant.

### Ethical considerations

Interviewers were trained and certificated in study procedures. Participants were identified though an ID number for confidentiality. All study procedures were revised and approved by the Research Ethics Committee from National Institute of Psychiatry “Ramón de la Fuente Muñiz”, Mexico City.

## Results

### Sociodemographic characteristics

Participants had an average age of 42.3 years (*sd* = 12) and 8.3 years of education (*sd* = 4), the intellectual disability group were younger (32.4 [*sd* = 10.1]) and had fewer years of education (5.4 [4.3]) when compared to other disability groups. Of the sample, 50.6% were men, however, in the mixed disability group there was a majority of women (54.7%). Almost all persons with intellectual disabilities (96.9%) reported have never been married compared to the visual impairment group, 55.7% reported being married or living in free union.

Approximately 64% of the sample is unemployed, the intellectual disability group has a higher prevalence (75%), followed by mixed (66.7%) and motor disability (62.7%). About the origin of the disability, 38.2% of the sample reported acquired it congenitally, being the intellectual disability group the one with the highest prevalence (85.6%) in comparison with the others groups. Likewise, almost half (45.8%) of the motor disability group reported that their disability was caused by an accident; the mixed disability group acquired it by a chronic disease (58.3%). The visual disability group reported accident or chronic diseases (37.7% in both groups) as the main cause of their disability. More than 70% of the total sample report required specialized services given their condition, and the majority considered that they have had access to them. Statistically significant differences were found in almost all variables, with the exception of sex and access to specialized services (see Table 1).

**TABLE 1:** Demographic characteristics of the sample by disability type.

	Motor *n* = 593		Visual *n* = 140		Intellectual *n* = 160		Mixed *n* = 203		Total *n* = 1,098		Statistical differences
	$\bar{\text{x}}$	(SD)	$\bar{\text{x}}$	(SD)	$\bar{\text{x}}$	(SD)	$\bar{\text{x}}$	(SD)	$\bar{\text{x}}$	(SD)	

Age (years)	44.6	(11.3)	44.5	(10.6)	32.4	(10.1)	41.6	(12.2)	42.3	(12)	*F*_(4,__1_,_093)_ = 39.0**
Education (years)	9.1	(3.6)	8.5	(3.7)	5.4	(4.3)	8.3	(4.1)	8.3	(4)	*F*_(4,__1_,_090)_ = 28.3**
	**n**	**(%)**	**n**	**(%)**	**n**	**(%)**	**n**	**(%)**	**n**	**(%)**	
		
**Sex**											*χ^2^*_(3)_ = 5.6
Male	297	(50.1)	74	(52.9)	92	(57.5)	92	(45.3)	555	(50.6)	
Female	296	(49.9)	66	(47.1)	68	(42.5)	111	(54.7)	541	(49.4)	
**Marital status**											*χ^2^*_(3)_ = 109.7**
Married/free union	261	(441)	78	(55.7)	5	(3.2)	79	(38.7)	423	(38.6)	
Never married	331	(55.9)	62	(44.4)	155	(96.9)	125	(61.4)	673	(61.4)	
**Employment**											*χ^2^*_(9)_ = 28.0**
Full time	68	(11.5)	25	(17.9)	12	(7.5)	19	(9.3)	124	(11.3)	
Part-time	123	(20.7)	33	(23.6)	15	(9.4)	36	(17.6)	207	(18.9)	
Student	27	(4.6)	6	(4.3)	13	(8.1)	13	(6.4)	59	(5.4)	
Unemployed	372	(62.7)	75	(53.5)	120	(75.0)	136	(66.7)	703	(64)	
**Disability Origin**											*χ^2^*_(3)_ = 198.5**
Congenital	151	(25.6)	41	(29.5)	137	(85.6)	88	(43.3)	417	(38.2)	
Acquired	438	(74.4)	98	(70.5)	23	(14.4)	115	(56.7)	674	(61.8)	
Chronic disease	153	(34.9)	37	(37.7)	4	(17.3)	67	(58.3)	261	(38.7)	
Accident	201	(45.8)	37	(37.7)	4	(17.3)	27	(23.5)	269	(39.9)	
Violence	7	(1.5)	9	(9.1)	1	(4.3)	2	(1.8)	19	(2.8)	
Substance use	1	(0.2)	3	(3)	0	(0)	0	(0)	4	(0.6)	
**Access to specialized services**											*χ^2^*_(3)_ = 1.1
Need	416	(70.4)	97	(69.3)	110	(68.8)	148	(72.9)	771	(70.5)	
Have access	285	(68.5)	62	(63.9)	77	(70)	103	(69.1)	527	(68.3)	

*p < 0.05, **p < 0.001.

### Prevalence of addictive disorders, psychiatric symptoms, suicidal behavior, and potentially traumatic events

In relation to substance use in the previous 30 days in the total sample, the average number of days of use was: 15.6 (*sd* = 13.6) days of tobacco use, 7.5 (*sd* = 10.6) of cocaine use and 6.6 (*sd* = 9.4) days of marihuana use; the group of visual disability consumes marijuana (22 days) and cocaine (15 days) more days; tobacco and alcohol consumption was similar in all groups.

As shown in Table 2, NUD was more prevalent in the visual (17.9%) and motor disability (13.8%) groups, finding statistically significant differences between groups comparison (χ^2^= 25.2, *gl* = 3, *p* ≤ 0.001). The presence of any AD (15% motor, 15% visual, and 10.3% in mixed disability) also shows significant differences. The intellectual disability group presented a higher proportion of MDD (22.5%), GAD and OPD (20.6% in both disorders), followed by the mixed disability group (18.1 and 14.2%, respectively). The MDD had a similar prevalence in all groups.

**TABLE 2:** Prevalence of addictive disorders, psychiatric disorders, suicidal behavior, and potentially traumatic events (PTEs) by disability type.

	Motor *n* = 593		Visual *n* = 140		Intellectual *n* = 160		Mixed n = 203		Total *n* = 1098		Statistical differences
	$\bar{\text{x}}$	(SD)	$\bar{\text{x}}$	(SD)	$\bar{\text{x}}$	(SD)	$\bar{\text{x}}$	(SD)	$\bar{\text{x}}$	(SD)	
**Days of substance use (past 30 days)**											
Nicotine	15.8	(14.2)	16.6	(12.7)	15.7	(12.4)	12.9	(12.1)	15.6	(13.6)	*F*_(3,__212)_ = 0.4
Alcohol	0.8	(1.4)	1.0	(1.1)	0.3	(0.4)	0.7	(0.8)	0.8	(1.3)	*F*_(3,__303)_ = 1.7
Marijuana	3.6	(6.4)	22.0	–	–	–	–	–	6.6	(9.4)	*F*_(1,__4)_ = 6.8
Cocaine	–	–	15.0	–	–	–	–	–	7.5	(10.6)	*F*_(1)_ = **----**

	**n**	**(%)**	**n**	**(%)**	**n**	**(%)**	**n**	**(%)**	**n**	**(%)**	
		
**Addictive disorders**											
NUD	82	(13.8)	25	(17.9)	5	(1.3)	13	(6.4)	125	(11.4)	χ^2^_(3)_ = 25.2**
AUD	14	(2.4)	5	(3.6)	3	(1.9)	5	(2.5)	27	(2.5)	χ^2^_(3)_ = 0.9
SUD	3	(0.5)	1	(0.7)	0	(0.0)	0	(0.0)	4	(0.4)	χ^2^_(3)_ = 2.1
GD	23	(3.9)	5	(3.6)	2	(1.3)	5	(2.5)	35	(3.2)	χ^2^_(3)_ = 3.2
Any AD	89	(89.0)	21	(15.0)	6	(3.8)	21	(10.3)	137	(12.5)	χ^2^_(3)_ = 16.3**
**Other psychiatric disorder**											
GAD	74	(12.5)	16	(11.4)	33	(20.6)	37	(18.1)	160	(14.6)	χ^2^_(3)_ = 9.6*
MDD	95	(16.0)	27	(19.3)	36	(22.5)	44	(21.6)	202	(18.4)	χ^2^_(3)_ = 6.2
Any OPD	71	(12.0)	17	(12.1)	33	(20.6)	29	(14.2)	150	(13.7)	χ^2^_(3)_ = 8.3*
**Suicidal behavior**											
Want to be dead	97	(16.4)	29	(2.7)	17	(1.6)	35	(3.2)	178	(16.3)	χ^2^_(3)_ = 8.9*
Thoughts 30 days	29	(4.9)	12	(8.6)	7	(4.4)	9	(4.4)	57	(5.2)	χ^2^_(3)_ = 3.8
Planning 30 days	13	(2.2)	4	(2.9)	7	(4.4)	3	(1.5)	27	(2.5)	χ^2^_(3)_ = 3.5
Attempts 30 days	9	(1.5)	4	(2.9)	2	(1.3)	3	(1.5)	18	(1.6)	χ^2^_(3)_ = 1.5
Attempts lifetime	90	(15.2)	25	(18.0)	10	(6.3)	28	(13.8)	153	(14.0)	χ^2^_(3)_ = 10.4*
**Potentially traumatic events**											
War zone combat	1	(0.1)	2	(1.4)	2	(1.2)	0	(0.0)	5	(.4)	χ^2^_(3)_ = 0.7
Verbal violence (infancy)	165	(28)	38	(27.5)	43	(27)	69	(34)	315	(28.9)	χ^2^_(3)_ = 3.2
Physical punishments (infancy)	79	(58.5)	21	(60)	17	(73.9)	26	(65)	143	(61.4)	χ^2^_(3)_ = 12.2*
Verbal violence	246	(41.6)	56	(40)	53	(33.5)	77	(38.1)	432	(39.7)	χ^2^_(3)_ = 3.6
Physical punishments	253	(42.8)	70	(50)	29	(18.4)	76	(37.4)	428	(39.2)	χ^2^_(3)_ = 39.1**
Serious accidents	244	(41.3)	42	(30)	34	(21.4)	70	(34.5)	390	(35.7)	χ^2^_(3)_ = 24.3**
Chronic disease	105	(17.8)	31	(22.1)	15	(9.5)	57	(28.2)	208	(19.1)	χ^2^_(3)_ = 21.8**
Death witness	188	(31.9)	49	(35)	28	(17.6)	55	(27.1)	320	(29.3)	χ^2^_(3)_ = 15.1**
Sexual abuse	60	(10.2)	25	(18)	14	(8.8)	23	(11.3)	122	(11.2)	χ^2^_(3)_ = 8.2*
Discrimination	310	(52.5)	72	(51.8)	85	(54.5)	96	(47.5)	563	(51.8)	χ^2^_(3)_ = 2.1

NUD, Nicotine use disorder; AUD, Alcohol use disorder; SUD, Substance use disorder; GD, Gambling disorder; AD, Addictive disorder; GAD, General Anxiety disorder; MDD, Mayor depressive disorder; OPD, Other psychiatric disorder. *p < 0.05, **p < 0.001.

Regarding suicidal behavior, 16.4% of people with motor disability had wished they had been dead in the last 30 days. The visual disability group had a higher prevalence of suicidal thoughts in the past 30 days (8.6%), however, there was not statistically significant differences. Lifetime suicide attempts are present in greater proportion in the visual disability group (18%) than motor disability (15.2%) and mixed (13.8%) groups with significant differences (χ^2^= 10.4, *gl* = 3, *p* ≤ 0.001).

The most prevalent PTEs were physical punishment in childhood (61.4%), followed by discrimination (51.8%), verbal violence (39.7%), physical punishment (39.2%), and serious accidents (35.7%). However, there are differences by groups. The intellectual disability group reported higher presence of physical punishment during childhood (73.9%), verbal violence (33.5%) and serious accidents (21.4%); Participants with motor disability had physical punishment during childhood (58.5%), physical punishment (42.8%), verbal violence (41.6%), and serious accidents (41.3%). People with visual disabilities suffered physical punishment during childhood (60%), physical punishment (50%), and verbal violence (40%), while those with mixed disabilities suffered physical punishment during childhood (65%), verbal violence (38.1%) and physical punishment (37.4%). In all groups, more than 50% had suffered discrimination. Statistically significant differences were found in almost all PTEs with the exception of verbal violence in childhood, verbal violence and discrimination.

### Relationship between potentially traumatic events and addictive disorders, psychiatric symptoms, and suicidal behavior

As shown in Table 3, verbal violence during childhood increases the risk of develop AUD (*OR* = 2.82, 95 % IC[1.22–6.61], *p* < 0.001), GAD (*OR* = 2.10, 95 % IC[1.45–3.03], *p* < 0.001), MDD (*OR* = 2.53, 95 % IC[1.80–3.56], *p* < 0.001), want to be dead (*OR* = 2.78, 95 % IC[1.96–3.93], *p* < 0.001), thoughts suicidal in the past 30 days (*OR* = 2.40, 95 % IC[1.36–4.27], *p* < 0.001), planning suicidal in the last 30 days (*OR* = 2.34, 95 % IC[1.04–5.40], *p* < 0.001), attempts suicidal in the past 30 days (*OR* = 3.92, 95 % IC[1.34–13.2], *p* < 0.001) and attempts suicidal lifetime (*OR* = 2.10, 95 % IC[1.43–3.07], *p* < 0.001). People who reported sexual abuse were more likely to present GAD (*OR* = 1.86, 95 % IC[1.18–2.90], *p* < 0.001), MDD (*OR* = 2.13, 95 % IC[1.38–3.28], *p* < 0.001), planning suicidal in the last 30 days (*OR* = 2.72, 95 % IC[1.13–6.35], *p* < 0.001), and attempts suicide lifetime (*OR* = 2.98, 95 % IC[1.91–4.59], *p* < 0.001).

**TABLE 3:** Odds ratio between potentially traumatic events (PTEs) by addictive disorder, psychiatric symptoms, and suicidal behavior.

	Addictive disorders			Psychiatric symptoms		Suicidal behavior				
	NUD	AUD	GD	GAD	MDD	Want to be dead	Thoughts 30 days	Planning 30 days	Attempts 30 days	Attempts lifetime

**Potentially traumatic events**	**Odds ratio [95% CI]**	**Odds ratio [95% CI]**	**Odds ratio [95% CI]**	**Odds ratio [95% CI]**	**Odds ratio [95% CI]**	**Odds ratio [95% CI]**	**Odds ratio [95% CI]**	**Odds ratio [95% CI]**	**Odds ratio [95% CI]**	**Odds ratio [95% CI]**
Verbal violence (childhood)	1.23 [.74–2.04]	2.82** [1.22–6.61]	1.92 [.92–4.05]	2.10** [1.45–3.03]	2.53** [1.80–3.56]	2.78** [1.96–3.93]	2.40** [1.36–4.27]	2.34** [1.04–5.40]	3.92** [1.34–13.2]	2.10** [1.43–3.07]
Physical punishments (childhood)	1.00 [.56–1.73]	1.27 [.51–3.07]	1.26 [.58–2.68]	1.19 [.79–1.78]	1.29 [.88–1.89]	1.20 [.81–1.76]	1.49 [.82–2.70]	1.31 [.56–3.05]	.97 [.31–2.88]	1.98** [1.32–2.95]
Current verbal violence	1.37 [.82–2.30]	1.26 [.51–3.10]	.60 [.26–1.34]	1.30 [.88–1.93]	1.05 [.72–1.51]	1.57** [1.01–2.27]	1.04 [.57–1.92]	1.17 [.48–2.87]	1.28 [.40–3.96]	1.62** [1.07–2.46]
Current physical punishments	.96 [.59–1.54]	.66 [.28–1.48]	2.26** [1.06–5.15]	1.21 [.84–1.73]	1.50** [1.07–2.10]	.98 [.69–1.37]	1.10 [.63–1.92]	1.28 [.57–2.93]	1.14 [.40–3.36]	1.30 [.88–1.90]
Serious accidents	2.22** [1.40–3.55]	1.97 [.90–4.35]	3.98** [1.88–9.18]	.82 [.57–1.17]	.95 [.68–1.32]	.91 [.64–1.27]	1.27 [.74–2.17]	1.76 [.82–3.86]	2.42 [.88–7.30]	1.28 [.88–1.86]
Chronic disease	.84 [.45–1.48]	.95 [.34–2.30]	1.25 [.55–2.65]	1.64** [1.11–2.39]	1.38 [.94–1.99]	1.51** [1.03–2.18]	.98 [.51–1.79]	.82 [.31–1.93]	1.95 [.67–5.29]	1.46 [.96–2.20]
Death witness	1.91** [1.20–3.00]	1.47 [.66–3.19]	1.92 [.97–3.82]	.86 [.59–1.25]	1.27 [.90–1.77]	1.08 [.76–1.52]	.83 [.46–1.45]	1.42 [.65–3.01]	.98 [.33–2.65]	1.28 [.87–1.86]
Sexual abuse	1.32 [.67–2.81]	1.06 [.27–1.30]	1.33 [.55–3.56]	1.86** [1.18–2.90]	2.13** [1.38–3.28]	1.11 [.70–1.73]	1.72 [.86–3.23]	2.72** [1.13–6.35]	2.67 [.87–7.86]	2.98** [1.91–4.59]
Discrimination	1.91 [1.20–3.00]	1.70 [.76–3.82]	1.02 [.47–2.13]	1.51** [1.05–2.18]	1.52* [1.09–2.14]	1.36 [.98–1.93]	2.35** [1.29–4.50]	2.09 [.89–5.52]	2.72 [.83–12.29]	2.24** [1.51–3.40]

NUD, Nicotine use disorder; AUD, Alcohol use disorder; GD, Gambling disorder; GAD, General anxiety disorder; MDD, Mayor depressive disorder. *p < 0.05, **p < 0.001.

Discrimination presents an increased risk to GAD (*OR* = 1.51, 95 % IC[1.05–2.18], *p* < 0.001), MDD (*OR* = 1.52, 95 % IC[1.09–2.14], *p* < 0.05), thoughts suicidal in the past 30 days (*OR* = 2.35, 95 % IC[1.29–4.50], *p* < 0.001) and attempts suicidal lifetime (*OR* = 2.24, 95 % IC[1.51–3.40], *p* < 0.001). PTEs minor prevalence was serious accidents with NUD (*OR* = 2.22, 95 % IC[1.40–3.55], *p* < 0.001) and GD (*OR* = 3.98, 95 % IC[1.88–9.18], *p* < 0.001). Chronic disease increases probabilities to GAD (*OR* = 1.64, 95 % IC[1.11–2.39], *p* < 0.001) and want to be dead (*OR* = 1.51 95 % IC[1.03–2.18], *p* < 0.001). Current verbal violence present an increased dead wish (*OR* = 1.57, 95 % IC[1.01–2.27], *p* < 0.001) and attempts suicidal lifetime (*OR* = 1.62, 95 % IC[1.07–2.46], *p* < 0.001).

## Discussion

The present study aimed to explore the association between PTEs, substance use, psychiatric symptoms, and suicidal behavior among IDW. A high prevalence of NUD was observed in tobacco users, the presence of GAD and suicidal behavioral were present in all disability groups, as well as the exposure to various PTEs during childhood and lifetime. It was found that people exposed to PTEs (verbal violence in childhood, sexual abuse, serious accidents, and discrimination) have a greater probability to develop GAD, MDD and suicidal behavior, mainly.

These results indicate that IWD have a high prevalence of NUD (11.4%) and days of use (15.6 [*sd* = 13.6]) in tobacco users in the last 30 days, in accordance with previous studies (Kerr et al., 2013) that point out that in IWD tobacco is the substance with the greatest impact. It was found out that GAD occurs mainly in individuals with motor and visual disability, a possible explanation is that people with motor disabilities acquired it (74.4%) mainly by some serious accident or a chronic disease (37.7% in both; Buist-Bouwman et al., 2006; Antunes et al., 2018). Previous research reported that people with disabilities have higher rates of lifetime suicidal ideation and attempts (Lund et al., 2016), in addition to the presence of depression symptoms (Lutz and Fiske, 2018), that can cause a perception of hopelessness toward the future or an improvement in life quality, originated by a sensation of unmet interpersonal needs or an unmet need for social competence (Van Orden et al., 2012) when the disability is mainly physical (Lund et al., 2016), findings similar to this study.

In accordance with previous studies (Focht-New et al., 2008; Smedema and Ebener, 2010), several traumatic events were found in every group. The most prevalent were physical punishment during childhood (Govindshenoy and Spencer, 2007; Leeb et al., 2012) and adulthood (Focht-New et al., 2008), serious accidents (Secretaria de desarrollo social [SEDESOL], 2016), chronic disease (Smedema and Ebener, 2010; World Health Organization [WHO], 2011) and witnessing the death of a person (familiar o stranger; Focht-New et al., 2008), suggesting that people with disabilities tend to present a higher proportion of adverse events than individuals without disabilities.

Verbal violence in childhood had a stronger relationship with almost all variables compared to the rest of the PTEs, as in previous research, childhood abuse is a strong predictor of problematic alcohol use, which may serve as a strategy for managing insecurities and anxiety disorders stemming from neglect and other sources of stress in childhood (Choenni et al., 2017; Bellis et al., 2018). Another cause reported in several studies and identified in the present research was that childhood abuse increases the likelihood of presenting mental health problems, such as mood disorders as is the case of MDD (Nelson et al., 2017; Gallo et al., 2018; Duncko et al., 2019), and GAD (Gallo et al., 2018; Guo et al., 2021), in addition to suicidal behavior (Norman et al., 2012; Khan et al., 2015; Angelakis et al., 2019), supporting the impact of the severity of various conditions suffered during childhood.

The results of this study, consistent with previous literature, suggest that sexual abuse was a predictor of the presence of MDD (Grollman, 2012; Kilpatrick and Taylor, 2018), GAD (Elias and Paradies, 2016), and suicidal behavior (Lund et al., 2016). Likewise, discrimination is considered a factor that often increases the risk of presenting mental health problems such as MDD and GAD (Pérez-Garín et al., 2018; Lund, 2021), as well as an increased risk of presenting suicide behaviors and attempts. This may be related to stigma, social isolation and victimization, situations that makes them feel trapped, hopeless, feel unprepared to work, low help-seeking, emotional stressed, and hopeless for the future, generating a deterioration in their life quality and mental health (Farrelly et al., 2015; Oexle et al., 2018), a condition that should be paid attention in psychological treatment schemes, as they tend to occur frequently.

An interesting finding of our study, was that serious accidents increased almost four times the risk of presenting GD, these are similar to another study, where they observed that exposure to traumatic events in childhood and adulthood generated a vulnerability to develop gambling problems (Scherrer et al., 2007; Luce et al., 2016; Buchanan et al., 2020), this may occur because individuals who develop GD tend to have poor coping mechanisms for dealing with stressful events, as well as poor emotional control and adequate regulation, which may hinder more effective solution-focused coping (Buchanan et al., 2020), increasing the problem gambling severity (Ronzitti et al., 2018) and frequent relapses (Oakes et al., 2012; Breen and Hing, 2014). Then, gambling is used as a coping mechanism for stress and negative mood states.

These data suggest that people with some types of disability are more likely to be exposed (perhaps more than the rest of the population) to PTEs, therefore they have a higher risk of presenting mental health problems; suggesting the need to increase social assistance programs, particularly those that have a direct impact on reducing discrimination and protecting the sexual integrity of these people. These findings suggesting the need for specific screening tools (Sheehan et al., 1998) and depth interviews (Spitzer et al., 1992; Kessler and Üstün, 2004) for an adequate identification of possible cases of some mental health problem. The evaluation must be carried out in primary health care centers; promoting the early detection of any possible mental disorder and provide referral to mental health centers, offering the opportunity for treatment for any psychiatric conditions, addictive behavior, and suicidal behavior, in order to have a significant impact in the mental health of people with disabilities (Iezzoni, 2011).

Several reports have established that IWDs require a broad spectrum of health services, from low-cost to complex and costly interventions caused by the comorbidity of mental and health conditions associated with disability (Merikangas et al., 2007; Sousa et al., 2009). The IWD and their families often incur in additional expenses, this may go toward health care services, assistive devices, costlier transportation options, heating, special diets, or personal assistance (World Health Organization [WHO], 2011). Various studies have been conducted in different countries to estimate the extra cost of disability, reporting that between 11 and 70% of the family income is destined to services that the IWDs need (Zaidi and Burchardt, 2005; Saunders, 2007), as well as 2–25% of the gross domestic product (GDP) in public programs, health services, rehabilitation, and social security benefits (World Health Organization [WHO], 2011).

Unfortunately, the need for services is almost twice than they can access to, people with disabilities require various specialized health services, they deal with poor accessibility to public and private facilities, non-inclusive means of transport, low pedagogical material and inadequate work or medical equipment, lack of adequate road for displacement, poor infrastructure that provides adequate care and personal barriers that prevent that the IWD look for treatment (Secretaria de desarrollo social [SEDESOL], 2016). The problematic in the need and access to specialized health services, is a finding reported in this study and in international reports (New Zealand Ministry of Health, 2004; Australian Institute of Health and Welfare, 2007; World Health Organization [WHO], 2011).

### Limitations/Strengths

This study has some limitations. First, people with communication disabilities were excluded, due to lack of resources or specialized personnel in the interpretation of signs, so the information of this group was not available. Second, people who were diagnosed with schizophrenia or bipolar disorder were excluded. Third, other metal disorders were not explored, to observe the comorbidity of different conditions in people with disabilities, considering the need to develop a broader study of mental disorders in IWD, using standardized diagnostic tools (CIDI or SCID) for a better diagnostic evaluation. An important strength to consider is that this is the first study carried out in Mexico focused on measuring multiple variables associated with mental health in IWD, using structured procedures and having field supervision by trained personnel in evaluation, to ensure the integrity of the data collected.

## Conclusion

People with disabilities are a group at-risk of different mental illnesses, which are derived by different causes or PTEs during childhood or adulthood; however, as they have limited access to specialized and individualized health services, it limits and sets back their attention and treatment, which impacts directly their quality of life. Among the governmental/institutional factors, there are not public policies to facilitate health services access offered by the state; there is a lack of funding for insurances to cover medical expenses, more specifically for rehabilitation and treatment services. Finally, there is a deficiency of general and specialized health services, as well as trained human resources to care for IWD; service hours are limited, and infrastructure is not enough and does not allow IWD to move easily inside the facilities.

## Data availability statement

The raw data supporting the conclusions of this article will be made available by the authors, without undue reservation.

## Ethics statement

The studies involving human participants were reviewed and approved by the Research Ethics Committee from National Institute of Psychiatry “Ramón de la Fuente Muñiz”, Mexico City. The patients/participants provided their written informed consent to participate in this study.

## Author contributions

RS-D participated in the implementation, collected the data phase, and conducted the data analysis. RM-N was the principal investigator of the project and developed the study protocol. AP-L participated in the implementation and collected the data phase. RS-A participated in the writing process and contributed as an expert. All authors contributed to the article and approved the submitted version.
